# Is Anorexia Nervosa a Disorder of the Self? A Psychological Approach

**DOI:** 10.3389/fpsyg.2016.00849

**Published:** 2016-06-14

**Authors:** Federico Amianto, Georg Northoff, Giovanni Abbate Daga, Secondo Fassino, Giorgio A. Tasca

**Affiliations:** ^1^Regional Expert Centre for Eating Disorders, Neurosciences Department, Psychiatry Section, University of TurinTurin, Italy; ^2^Mind, Brain Imaging and Neuroethics Research Unit, The Royal’s Institute of Mental Health Research, University of Ottawa, OttawaON, Canada; ^3^Psychology, The Ottawa Hospital, University of Ottawa, OttawaON, Canada

**Keywords:** anorexia nervosa, eating disorders, attachment, self, neurobiological pathway

## Abstract

The debate concerning the pathogenesis and the maintaining factors of eating disorders, anorexia nervosa in particular, is ongoing especially since therapeutic interventions do not result in satisfactory and enduring rates of remission. This paper presents a model for the pathogenesis of eating disorders, based on the hypothesis of a deficiency in the development of the self. We present the theory in light of new evidence concerning the role of attachment insecurity in the development and maintenance of eating disorders. In particular, we define the self in eating disorders in a comprehensive way by taking into account recent evidence from experimental psychology and neurobiology. The paper considers the development of the self in terms of its synchronic (i.e., experienced in the moment) and diachronic (i.e., experienced as continuous over time) aspects. Both synchronic and diachronic aspects of the self are relevant to the expression of eating disorder symptoms. Further, the maturation of the self is interwoven with the development of attachment functioning from infancy to adolescence. This interplay between these developmental processes of the self and of attachment could be crucial in understanding the pathogenesis of eating disorders. The final part of the paper suggests a neurobiological link between the theory of the self in the eating disorders and the spatiotemporal functioning of the brain. Disturbances in spatiotemporal functioning may represent the neurobiological pathway by which deficiencies in the self is related to attachment functions in individuals with eating disorders.

## The Self in Anorexia Nervosa

Most theories on the pathogenesis and maintenance of eating disorders, including AN, have been based on cognitive-behavioral models that focus on maintenance factors such as high level of need to control eating, judging self-worth based on shape and weight, and dietary restraint (Fairburn et al., 2003, 2005; Murphy et al., 2010). Yet such models do not provide a pathogenic explanation including the experiential or phenomenological and interpersonal aspects of the disorder that are prominent in the clinical presentation and in clinical interactions. That is, those with AN often report: profound disconnection with their own emotions and body sensations (Skårderud, 2007), difficulty in understanding their own and others’ internal experiences (Rothschild-Yakar et al., 2013; Tapajóz Pereira de Sampaio et al., 2013), and problems in constructing a personal narrative over time, especially related to the disorder (Oldershaw et al., 2011; Treasure, 2013; Lang et al., 2014). Further, in reaction to such patients, clinicians may experience troubling emotional responses including a sense of helplessness and anger (Vitousek et al., 1998; Satir et al., 2009; Fassino and Abbate-Daga, 2013).

Given the limitations of current prominent theories of AN, and considering the context of the phenomenological and interpersonal nature of the disorder, we propose a theory of the self in AN in which the self represents the organizing function of the mind that when disturbed, will lead to and maintain the disorder. A fundamental tenet of this approach is that AN symptoms are a way of managing painful internal experiences related to a deficit of the self (Tasca and Balfour, 2014a; Williams et al., 2015).

In this paper we outline a theoretical model of eating disorders, and of AN in particular, in which we illustrate our conceptualization of the self, and define AN as a disorder of the self. We argue that deficits in the self are the basis of eating disorder psychopathology, and that these deficits can be understood at the neurobiological level and at the clinical-developmental level. The main focus of our model is on anorexia nervosa because in which a deficit of the self is more extensive and deep; nevertheless it is possible to extend our model to the other eating disorders. For instance, binge eating, which may be a means of regulating negative emotions in those with bulimia nervosa or binge-eating disorder can derive from a deficit of the self. Such a deficit in these individuals is related to problems in attachment and thus to emotion dysregulation that may be a precursor to binge eating (Tasca et al., 2009a). As a broad guiding principle, we will use attachment theory (Bowlby, 1969, 1973, 1980) to anchor our presentation because it is an extensive and well-tested model of adult development and psychopathology (Mikulincer and Shaver, 2010). We will briefly discuss treatment and research implications that are informed by our theoretical model.

## A Definition of the Self

What exactly is the self? The self may be understood as an integrative structure of the mind that organizes and coordinates different functions (affective, cognitive, social, sensorimotor, and vegetative) with regard to interoceptive and exteroceptive stimuli from one’s own body and the environment (Northoff, 2013). In this model, the self is the cognitive, conative, and affective representation of one’s subjective experience and one’s identity. Thus, the self plays an integral part in human motivation, cognition, affect, and social identity.

The self also implies a temporal concept. It extends across time from the past over the present and to the future. In contrast to the concept of identity, which describes the diachronic sameness of a person over time, the concept of self is synchronic, that is, it concerns the experience of an individual as him or herself, and allows him or her to attribute specific experiences, persons, or objects at a particular point in time (Doering et al., 2012). This entails a self-specific organization of both neural and psychological activity (Northoff, 2015a,b). Alterations in the self-specific organization may consequently have an impact on all subsequent dependent functions, such as abnormal changes in affective, cognitive, sensorimotor, vegetative, and social functions.

## Basic Deficit of the Self in Anorexia Nervosa

A number of years ago (Bruch, 1982) proposed that at the root of AN was fundamentally a deficit of the self. As we suggested above, the integrative function of the self (i.e., its ability to integrate cognitive, affective, and conative functions) in those with AN is compromised. Bruch (1982) argued that individuals with AN function with a “false-self,” which implies that these individuals may not discriminate between their own and their caregivers’ expectations and needs (Winnicott, 1964). More current conceptualizations indicate that individuals with an eating disorder are not able to discriminate between the sensations pertaining their body (e.g., hunger) and their emotions (e.g., anger; Skårderud, 2007). More importantly, and of particular relevance to our presentation, is the likelihood that their experience of their body is not integrated into their self. Even though patients with AN do not perceive their body as sharply extraneous, as sometimes happens in schizophrenia (Northoff, 2015a), they do appear to maintain an attitude of “objectification” toward their body, as if their body does not pertain to their self. The body is no longer experienced in a subjective way as “my” body, and thus as personal or self-related. Instead, the body is a mere object that is impersonal or non-self-related, with no special relationship to the self (Greenleaf and McGreer, 2006; Fitzsimmons-Craft et al., 2011; Eshkevari et al., 2014). This is consistent with theories that suggest that physiological responses in the body are the origin of emotions. Such theories include both neuroscientific and philosophical concepts of embodiment of emotional feelings (Northoff, 2012). Hence, those with anorexia nervosa may not perceive the physiological correlates of the body, and thus they may not experience, identify and express related emotions.

Bruch (1982) also suggested that the diachronic function of the self (i.e., the experience of a consistent self over time indicated by one’s identity) is interrupted. That is, the adolescent with AN has difficulty to consider their future and to perceive and integrate their past into a current narrative of the self. The impact of this deficit is that such individuals with AN will have problems integrating their own internal experiences within a meaningful narrative of the self as persisting across time. Such problems will result in an unstable identity. The unstable sense of self and identity weakens related functions like self-esteem, emotion regulation, and interpersonal effectiveness. Contemporary psychoanalytic writers have expanded on these concepts by discussing how problems in early caregiver-child relationships may lead to basic difficulties with identifying feelings and integrating them into a sense of self (Granieri and Schimmenti, 2014). Such difficulties may also be associated with problems in using symbols to represent internal states, thus hampering the ability to construct meaning (Lavender and Freedman, 2002).

A pervasive weakness of the integrative functions of the self may explain why certain personality traits that are common to AN become rigid, such as high harm avoidance, low self-directedness (Fassino et al., 2002, 2013), and perfectionism (Wade et al., 2008; Hurst and Zimmer-Gembeck, 2015). These traits become stable during adolescence, play a key role in the maintenance of AN, and often remain even after recovery from the disorder (Wagner et al., 2006). Moreover, a theory of deficits in the self’s functions is consistent with a multifactorial model of the development and maintenance of AN, since the self integrates various aspects of mental functioning.

Deficits of the self in those with AN likely have their origins in attachment insecurity (Blatt and Blass, 1990; Tasca and Balfour, 2014a; Gander et al., 2015), and also in other sociocultural factors (i.e., pressures to be thin, and internalization of the thin ideal), non-shared life events (e.g., traumatic experiences), and genetic vulnerabilities to being underweight (Klump et al., 2009). Before discussing attachment theory in more detail as a framework for our model of the self in AN, we review theories and research on deficits of the self in AN. We argue that problematic functions of the self result in difficulties in integrating current self-image and in relationships with others, which may generate feelings of inadequacy and social insecurity. These feelings of inadequacy and insecurity are among the most enduring maintaining factors of eating disorders (Fairburn et al., 2003; Arcelus et al., 2013).

## The Relational Self and Attachment in Eating Disorders

Healthy individuals perceive a clear distinction between their body, their emotions, and relationships, and those that pertain to others (Decety and Sommerville, 2003). The organizing function of the self permits one to perceive what is one’s own and what is the “other” (Doering et al., 2012). In this sense, the self plays a relational function because it defines and differentiates the participants of a relationship, since no true “relationship” exists without a differentiation between self and other (Erikson, 1968). Along similar lines, Schore (1994) discusses the self as developing within the context of an environment (i.e., parental caregiving and attachment), and as experience-dependent.

The relational self begins in infancy during the process of differentiation (Mahler, 1963). Blatt and Blass (1990) reformulated Erikson’s (1968) developmental model to provide a useful developmental framework in which both the relatedness and the self-definition lines of development work in a dialectic process across developmental phases. For example: (i) developing a sense of trust in a caregiver during infancy (a relatedness function) (ii) allows one to achieve adequate autonomy of the self as a toddler (i.e., a self-definition element), which in turn (iii) creates the conditions for becoming a differentiated autonomous self during the latency period (i.e., self-definition), that later (iv) gives way to mutuality in early adolescence (i.e., a relatedness function). Ideally, these developmental processes become integrated in (v) a clearly defined identity during late adolescence or early adulthood.

A secure attachment with a caregiver in infancy provide the context within which these developmental lines and processes facilitate the emergence of a relational and autonomous self. Difficulties with attachment security among those with eating disorders may be associated with a history of trauma in childhood and adolescence, and may result in heighted eating disorder symptoms, alexithymia, and shame (Franzoni et al., 2013; Tasca et al., 2013).

Bowlby (1969) described the attachment behavioral system (e.g., crying, reaching, crawling, and eye gazing) as an inborn process that increases proximity between an infant and caregiver especially during times of threat or stress. Caregivers may respond in various ways including by being optimally available, unavailable, inconsistent, or frightening. The repeated interactions between infant and caregivers that are set in motion by infant attachment behaviors become encoded in the implicit memory system of the child as internal working models of attachment that act as schemata for future relationships (Siegel, 1999). Attachment patterns are often stable from infancy to adulthood, though changes are possible due to life events or changing interactions with attachment figures in adolescence and adulthood (Pinquart et al., 2013). Individual differences in organization of attachment behavior are to some extent related to the responses of attachment figures. Children, adolescents, or adults who experience caregivers or attachment figures as not providing adequate security, emotional availability, and attunement may develop maladaptive dependency or detachment as secondary defensive strategies (i.e., attachment anxiety/preoccupation or attachment avoidance/dismissing, respectively) when faced with threat or stress.

The Adult Attachment Interview (AAI; George et al., 1985; Main et al., 2003) is the gold standard for investigating the attachment system in adults. The AAI and its concepts are important for understanding eating disorders because the AAI primarily assesses attachment states of mind. Of particular relevance to this presentation, the AAI allows one to code two scales specifically related to the self and its functions: Coherence of Mind and Reflective Functioning, each of which is described below. The AAI is a clinical, semi-structured interview focusing on the interviewee’s current mental representations of early attachment experiences (including the loss of loved ones and other traumatic experiences), and their influence and possible impacts on adult personality and behavior. Both the coherence of the interviewee’s memories reflected in their narratives, and the quality of their collaboration with the interviewer serve to inform one about the interviewee’s internal working models of attachment.

Among the AAI scales, the Coherence of Mind scale is the most relevant indicator of the speaker’s state of mind with respect to attachment (Main, 2000). Coherence of mind is characterized by the interviewee’s ability to provide a relevant and cohesive narrative about their attachment experiences that is collaborative with the interviewer. The quality of coherent narratives is not diminished by: (i) intrusions of current emotions about the attachment relationships, or (ii) a defensive memory structure that results in very brief and incomplete recollections. Thus, coherence of mind may be related to the “narrative self” that may indicate the integration and stability of the self over time (Jacobs et al., 2003). Poor coherence of mind represents a specific difficulty in creating a personal narrative of one’s own psychological and personal development. As such, adequate coherence of mind may be an indicator of the quality of the diachronic nature of the self, that is, an indicator of the stability of the self over time, and thus of the coherence of the interviewee’s identity. In two case presentations, Tasca and Balfour (2014a) argued that patients with eating disorders often show low coherence of mind when they report their life experiences, both in the clinical interview and in the AAI transcripts. Also, researchers indicate low levels of coherence of mind among individuals with eating disorders, including those with AN (Ward et al., 2000; Fonagy and Target, 2006).

## Reflective Functioning and the Self

Reflective functioning, which can be rated in AAI transcripts is an indicator of mentalization and is related to theory of mind (Fonagy and Target, 2006). Interviewees’ responses to AAI demand questions such as: “why do you think your parents behaved the way they did during your childhood?” and “how do you think your childhood affected your adult personality?” are rated for the individual’s ability to understand their own mental states and the mental states of others. As such, reflective functioning is necessary for accurate empathy and to respond appropriately (i.e., to understand others’ mental states), and may be representative of a well-developed autonomous self (i.e., the ability to appreciate that one’s own mental state is unique from yet related to others’). Reflective functioning may be a proxy for the synchronous experience of the self (in contrast to the diachronic nature of identity indicated by coherence of mind). That is, reflective functioning may be an indicator of one’s sense of self as experienced in the moment and as separate but related to others. Research on eating disorders indicate that those with AN have particularly low scores on reflective functioning even compared to those with other psychiatric disorders (Fonagy et al., 1996).

Attachment insecurity, associated with low coherence of mind and low reflective functioning, can be characterized as anxious (or preoccupied) or avoidant (or dismissing). Attachment anxiety is associated with a maladaptive upregulation of the emotional system, and a preoccupation with loss or abandonment in relationships. Attachment avoidance is associated with a maladaptive down regulation of emotions, and a tendency to dismiss the importance of relationships. Both attachment anxiety and avoidance are associated with increased psychopathology and poorer treatment outcomes (Tasca and Balfour, 2014b). The clinical relevance of attachment dynamics for individuals with eating disorders is underlined by the association between greater attachment anxiety and avoidance with negative affect, body dissatisfaction, and eating disorder symptoms (Tasca et al., 2006, 2009a; Troisi et al., 2006; Illing et al., 2010). Moreover, notwithstanding the relevance of some personality traits for the expression of eating disorder symptoms (Fassino et al., 2002), attachment insecurity is related to eating psychopathology independent of personality characteristics (Abbate-Daga et al., 2010; Amianto et al., 2011). Nevertheless, substantial evidence of the specific relationship between attachment style, eating disorder diagnosis, and eating disorder psychopathology is still lacking, and more research is needed in this field (Tasca and Balfour, 2014b).

## Attachment and Development Stages Related to the Self in Eating Disorders

We now turn to describing in more detail a developmental model, and how problems in individual development are associated with deficits in the functioning of the self among those with eating disorders. In this section, we will use the developmental model articulated by Blatt and Blass (1990). As indicated, Blatt and Blass described a dialectic process across time in which relatedness-based and self definition-based developmental lines evolve mutually so that functions of the self, including identity, can form cohesively within the individual. Rather than focus on each of Blatt and Blass’s stages separately, we will contain our discussion to severe failure in the development of the self in eating disorders by focusing on three broadly defined lines or phases in childhood through adolescence: (i) the self definition developmental line, (ii) the relatedness developmental line, and (iii) identity development in adolescence as the culmination of the two developmental lines.

### First Stage: Self Definition and Body

As indicated, the first developmental line we will discuss is associated with self definition in childhood. This period and developmental line is crucial for the experience of the “body self” (Winnicott, 1964). The high level of difficulty individuals with eating disorders have relating to their body may be associated with a failure in this separation-individuation line of development (Blatt and Blass, 1990). Children experience and internalize a progressive distinction between their body and their mothers’ body. During this period of childhood, the “body self” acts as an objective reference to the emerging and autonomous self of the child. Two components of this developmental process include: the establishment of a gratifying involvement with the caregiver, and the eventual experience of incompatibility of the gratifying involvement with the development of a differentiated and autonomous self (Blatt and Blass, 1990). The ability of the caregiver to recognize the needs of the infant as differentiated from the caregiver’s self is crucial for the child to experience a distinction between his or her body from that of the caregiver. In this sense, it may be that the child needs to develop a sort of attachment to his or her own body, which is facilitated by the relationship with the caregiver who also recognizes his or her body as separate. Failing that, the child may perceive their body as if it was an object pertaining the external world instead of a substantial part of their self. A related phenomenon is Winnicott’s notion of the “false self,” which is a condition that is common in early childhood in which children perceive thoughts or desires of their caregivers as their own because they cannot distinguish them. Similarly, the work of Schimmenti and Caretti (2016) illustrates how neglectful or abusive caregiver-child relationships can lead to problems with relatedness and self-definition in the child. These involve multiple disconnections at the level of the self and relationships, which has implications for the experience of one’s body.

Among those with eating disorders, Stein and Corte (2003, 2007) found a particular conflation of self-esteem as body esteem. They argued that individuals with an eating disorder use their body-image as a proxy for their self-concept. This may be due to a deficit in the development of the self-definition. A negative body image and a high number of negative self-schemas can be understood as the expression of internalized aspects of self that may set the stage for the subsequent development of an eating disorder (Stein and Corte, 2003). In one study, self-schema as “fat” mediated the relationship between overall self-concept and eating disordered attitudes and behaviors in women with bulimia nervosa and AN (Stein and Corte, 2007). Thus, a deficit in the development of the self caused by problems with body-self definition may represent a core element in the emergence of eating disorder psychopathology.

### Second Stage: Relatedness and Others

The second line of development in which attachment influences the functioning of the self in childhood is the relatedness line of development (Blatt and Blass, 1990). As indicated, in order to activate attachment and caring behaviors from caregivers, children enact proximity-seeking behaviors. The interactions with caregivers that are set in motion by proximity seeking are encoded into the implicit memory system as internal working models, which contribute to the development of the self. Experiences of trust between infant and caregiver and of mutuality later in childhood are necessary for attachment security, which is necessary for adequate affect regulation and the development of a cohesive identity. As we discussed previously, attachment concepts of coherence of mind and reflective functioning represent the diachronic and synchronous nature of the self, respectively. We argue that deficits in these areas lead to problems with a coherent self narrative (i.e., a self that is not experienced as stable over time, which leads to heightened insecurity), and with difficulties understanding one’s own mental states and appreciating mental states in others (i.e., leading to affect instability and deficits in empathy).

Children who experience their caregivers as emotionally available and loving develop an internal working model of the self as loved and valued, and develop a model of others as loving and safe (Bowlby, 1973). When children experience their caregivers as inconsistent or unpredictable, neglectful, or rejecting children may develop attachment insecurity. This leads to a model of the self as unlovable, and a model of others as unloving or rejecting (Bartholomew and Horowitz, 1991; Ward et al., 2000). Accordingly, the experience of the child as valuable, worthwhile, and lovable in the context of the relationship with a caregiver creates a positive cognitive set about the self that is carried into adolescence and adulthood. Instead, insecure attachment is associated with negative views of the self and low self-esteem (Sharpe et al., 1998; Troisi et al., 2006), which in turn is a risk factor and a maintenance factor for eating disorders (Fairburn et al., 2003, 2005).

### Third Stage: Identity and Adolescence

Finally we discuss the “identity” phase of development as described by Blatt and Blass (1990). Identity as indicated by Erikson (1968) is a process of defining a unified self that is both separate from others without a sense of alienation, and connected to others without role confusion. In Blatt and Blass’ model, identity is the confluence of the self-definition and relatedness lines of development. Identity development typically takes place throughout adolescence and is a function of evolving attachment relationships with parents, emerging important relationships with peers, as well as the ongoing dialectic of self-definition and relatedness.

The ability to develop a cohesive identity is in part dependent on early attachment bonds with primary caregivers (Jacobs et al., 2003). The attachment to a caregiver thus remains essential during adolescence. A secure attachment pattern encourages individuals to look for intimacy, protection, and dependence from caregivers as a regulatory function in response to environmental stressors (Kenny and Hart, 1992). However, during late childhood and early adolescence, peer relationships begin to take more central roles in the development of self concept and self-esteem. In the course of preadolescence and adolescence, a heightened sensitivity to social and relational messages produces the need among adolescents to integrate new experiences, thoughts, and behaviors in relation to the self. The shift of emphasis from parental to peer relationships can put a strain on attachment relationships with primary caregivers, thus requiring adaptation in these attachment relationships.

Adolescence is also a time when changes occur to one’s body related to puberty, and concurrent structural changes that occur with maturation of the brain conditioned by sex hormones (Herting et al., 2014). The result is a challenge to the emerging identity and to the adolescent’s ability to manage and regulate their emotions.

The availability of secure attachment bonds in adolescence promotes the regulation of the individuals’ emotions. This is crucial since the internalization of a positive self-concept implies the ability to be aware of one’s emotions, to experience strong emotions without disruption, and to engage in a thoughtful examination of situations from a state of calm reflection and reasoning (Skowron and Dendy, 2004). As a consequence, a cohesive identity fostered by secure attachment promotes the active exploration and mastery of the environment, which is necessary for an individual to develop autonomous functions and intimacy in adulthood. Failure to experience one’s self as socially appreciated and lovable produces a specific vulnerability to disordered eating in adolescents (Arcelus et al., 2013).

An incohesive identity in those with an eating disorder likely results in them feeling incompetent or inadequate. Stein and Corte (2007) found that women with bulimia nervosa and AN had fewer positive self-schemas, more negative self-schemas, and higher interrelatedness among their self-conceptions compared to a control group. In conditions of relational stress, individuals with a frail self or incohesive identity may activate secondary attachment behaviors associated with preoccupied or dismissing attachments. These individuals may try to maintain a strong dependence with caregivers, or keep a maladaptive distance with respect to caregivers (Tasca and Balfour, 2014a). For example, Gordon (2000) found that individuals with bulimia nervosa do not feel that they have the capacity to be in a relationship while remaining autonomous. Individuals with an eating disorder may have a difficult time in conceiving their personal value independent of caregivers (Cozzi and Ostuzzi, 2007). Research evidence suggests that secure attachment to parents, with a balance between encouraging of autonomy while emphasizing connectedness, enables adolescents to develop a clear identity and a positive self-concept (Holmbeck and Wandrei, 1993; Deci and Ryan, 1995).

As indicated earlier, the attachment concept of coherence of mind (i.e., the ability to construct a coherent narrative of attachment relationships and, therefore, of the self; Main et al., 2003) is indicative of a cohesive identity (Doering et al., 2012). Cohesive identity is generally associated with emotional health and well-being, whereas instability is related to fragile self-esteem (Kernis, 2002). Research has indicated that poor self-concept (i.e., low self-esteem, self-confidence, and stability of the self) has a direct negative impact on the development of a stable identity among those with eating disorders (Tasca et al., 2009b). Further, there is growing evidence that coherence of mind is compromised among those with an eating disorder (Fonagy et al., 1996; Pedersen et al., 2012).

Individuals with eating disorders experience a deep discomfort due to the lacking sense of the self, which is based on their inability to perceive and integrate their body image and physiological responses, to regulate their emotions, and to experience a diachronic sense of their self. They organize their behavioral responses to control and reduce their deep discomfort using the management of eating behaviors (Skårderud, 2007). This may result in eating disorder symptoms, as described by cognitive-behavioral, attachment, and psychodynamic theorists (Fairburn et al., 2003; Fassino and Abbate-Daga, 2013; Tasca and Balfour, 2014a,b).

## Outlook – Self and Brain in Anorexia Nervosa

To summarize, we argue that there is value in understanding eating disorders, especially AN, as a disorder of the self and its functions. Previous studies found that patients with AN have functional and structural alterations in a wide network of brain areas, including the precuneus, DLPFC, cingulate cortex, insulae, temporal poles, thalamus, hypothalamus, caudate nucleus (Amianto et al., 2013; Cowdrey et al., 2014), and hippocampus-amygdala complex (Wittman et al., 2010). These brain alterations may occur at an early stage of the disorder and may overlap with the brain circuits related to attachment functions (Riem et al., 2012), which may be associated with the onset of the disorder.

The self can be seen in term of its diachronic nature and as a synchronous experience. Recent research in neurobiology has found that both diachronous and synchronous aspects of self are represented in the temporal-spatial characteristics of the resting state of the brain (Northoff, 2015a,b), and both of which are likely disturbed in AN. Recent data showed that self-related processing strongly overlaps with and is predicted by the resting state activity (Qin and Northoff, 2011; Whitfield-Gabrieli et al., 2011; Bai et al., 2016; Huang et al., 2016). This seems to be especially the case in midline structure as the core part of the default-mode network (DMN). Since various resting state networks including sensorimotor networks and DMN are altered in eating disorders, these may be closely related to the above described alterations in the self. This remains to be shown in the future, however.

With regard to diachronic functions and the self, one can argue that phenomenologically, individuals with AN have a difficult time integrating past and present into a coherent identity. In attachment terms, these problems are indicated by the low coherence of mind as assessed by the AAI (i.e., in problems with constructing a coherent narrative about attachment relationships). Neurobiologically, this may be evidence of what Northoff (2015a) called temporal dysfunction (see Northoff, 2015b for details) in the resting state of the brain, thus disrupting the perception of time and the integration of the self across past and present experiences.

With regard to synchronous functions of the self, individuals with AN appear to have difficulty in integrating current self functions into a synchronous experience so that the self and other are not well distinguished or integrated. In attachment terms one sees in these patients low reflective functioning and mentalization likely due to a disruption in the ability to appreciate the self and other as separate but related mental states. This in part may underlie the experience of body image distortion and body dissatisfaction. In neurobiological terms, this problem may be indicative of Northoff’s (2015b) notion of spatial dysfunction in the brain’s resting state, in which there is a maladaptive and almost exclusive focus on the self and perhaps a misperception of the body as not part of the self.

Clinically, our attachment-based model of the self in AN implies that clinicians must provide a therapeutic environment that aims to provide secure base (Bowlby, 1988) from which patients with AN can learn to develop affect regulation abilities, reflective functioning related to one’s own and others’ mental states, and a coherent narrative of the self over time. The relevance of attachment dynamics in the development of the sense of self in those with eating disorders suggests that systemic and multidisciplinary approaches to treating eating disorders and their families (e.g., Lock and Le Grange, 2015) may be necessary to facilitate the development of the self. Such approaches may help to overcome the temperament and neurobiological distortions associated with attachment insecurity and problems with the self (Lask, 2009; Treasure, 2013; Kaye et al., 2015; Abbate-Daga et al., 2016). A detailed discussion of these treatment approached goes beyond the aims of the present review.

In terms of research paradigms, our model suggests that those with AN will show disturbances in the brain’s resting state, and that these may be specifically associated with spatial functioning possibly related to experiencing one’s own body as an integrated aspect of the self, and temporal functioning possibly related to integrating the self in a coherent narrative over time.

## Author Contributions

FA contributed to the ideation of the review, to the search of literature, wrote the paper, translated the paper in English Language, revised the drafts of the paper. GN contributed to the ideation of the review, to the search of literature, wrote parts of the paper and summarized it, revised the English Language, revised the drafts of the paper. GAD contributed to the ideation of the review, and revised the drafts of the paper. SF contributed to the ideation of the review, to the search of literature, revised the drafts of the paper. GT contributed to the ideation of the review, to the search of literature, wrote parts of the paper, revised the English Language, revised the drafts of the paper.

## Conflict of Interest Statement

The handling Editor declared a shared affiliation, though no other collaboration, with several of the authors SF, GAD and FA, and states that the process nevertheless met the standards of a fair and objective review. The rest of the authors declare that the research was conducted in the absence of any commercial or financial relationships that could be construed as a potential conflict of interest.
